# Advances in biomimetic collagen mineralisation and future approaches to bone tissue engineering

**DOI:** 10.1002/bip.23527

**Published:** 2022-11-29

**Authors:** Michael Eugene Doyle, Kenny Dalgarno, Enrico Masoero, Ana Marina Ferreira

**Affiliations:** ^1^ School of Engineering Newcastle University Newcastle upon Tyne UK; ^2^ School of Engineering Cardiff University Cardiff UK

**Keywords:** apatite, biomimetic, bone tissue engineering, collagen, mineralisation

## Abstract

With an ageing world population and ~20% of adults in Europe being affected by bone diseases, there is an urgent need to develop advanced regenerative approaches and biomaterials capable to facilitate tissue regeneration while providing an adequate microenvironment for cells to thrive. As the main components of bone are collagen and apatite mineral, scientists in the tissue engineering field have attempted in combining these materials by using different biomimetic approaches to favour bone repair. Still, an ideal bone analogue capable of mimicking the distinct properties (i.e., mechanical properties, degradation rate, porosity, etc.) of cancellous bone is to be developed. This review seeks to sum up the current understanding of bone tissue mineralisation and structure while providing a critical outlook on the existing biomimetic strategies of mineralising collagen for bone tissue engineering applications, highlighting where gaps in knowledge exist.

## Graphical abstract

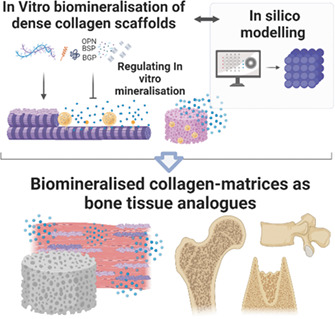

## 1 INTRODUCTION

Most conventional strategies for the repair of damaged bone incur further damage to already compromised tissues throughout their use. For instance, the implant site must be reshaped via the removal of bone tissue to accommodate a standardised component, this occurs again for each revision surgery required for fitting the implant. In addition, while the average service life of polymeric, ceramic, and metal‐based implants is improving (up to 58% of total hip replacements are reported to last up to 25 years as of 2019^[^ ^1^ ^]^), particles released through implant wear stimulate the action of macrophages which increase inflammatory response and stimulate the resorbing action of osteoclasts, whilst suppressing the differentiation of osteoblasts.^[^ ^2^ ^]^ In the case of static implants, Wolff's law dictates that the contrast in mechanical properties between the implant and host tissue induces disproportionate resorption of bone.^[^ ^3^ ^]^ These factors act in concert, resulting in an overall loss of bone throughout implant use in load bearing and articulating applications.^[^ ^4^ ^]^ Ultimately, the more closely matched a repair substrate is with the surrounding tissue, the less likely these issues are to arrive. In recent years, myriad hybrid materials (as compiled by Griffanti et al.^[^ ^5^ ^]^) have been developed which contain key components of bone to permit better integration along with synthetic components for mechanical/structural characteristics, none have attained the goal of synthesizing comprehensive *in vitro* bone substitutes. To manufacture biomimetic bone most aptly, it is essential that one possess a thorough understanding of not only the composition of bone and its sub‐components but also the nature of their assembly and evolution. This review aims to highlight the existing knowledge around the development of bone in the human body while providing a critical outlook on the strategies of mineralising collagen for bone tissue engineering applications, discussing controversies and knowledge gaps in this field. This review contributes to the field of bone tissue engineering by elucidating commonalities between seemingly disparate *in vitro* methodologies for synthesizing bone and establishing connections between them and *in vivo* processes. Below is a brief compilation of established state‐of‐the‐art knowledge surrounding *in vivo* bone development and composition.

### 1.1 Underpinning fundamental knowledge of bone tissues

In bone tissue engineering, scaffolds play a key role in housing bone cells and facilitating their biological functions. Therefore, a comprehensive understanding of biological mechanisms related to bone development is needed to inform engineers in their approaches to promoting tissue repair through biomimetic scaffold design. Bone mineralisation has been described as a matrix‐mediated process, in which type I collagen secreted by cells to constitute the native extracellular matrix (ECM) has the ability to modulate the nucleation and growth of mineral throughout the matrix.^[^ ^6^ ^]^ The mineral being carbonate substituted hydroxyapatite (HA), a phase of Calcium Phosphate (Ca/PO) [Ca_10_(PO_4_)_6_(OH)_2_]; it is complex and features a varied composition, Von Euw et al.^[^ ^7^ ^]^ reported that the average ionic composition of HA surface from 2‐year‐old sheep bone was Ca_7.5_(PO_4_)_2.8_(HPO_4_)_2.6_(CO_3_)_0.6_(OH)_0.2_. A ‘brick and mortar’ analogy has been proposed for visualising the interplay between these two essential components. The ‘bricks’ are an ordered network of crosslinked collagen molecules with a regular ‘D‐spacing’ arrangement, and the ‘mortar’ is a composite mixture of amorphous calcium phosphate (ACP) phases which reside in the gaps between collagen molecules.^[^ ^8^ ^]^ The mineral content by weight (wt%) within the ECM of bone is approximately 67%^[^ ^9^ ^]^ and typically around 32%^[^ ^10^ ^]^ for bone as a whole. Beyond these most prominent constituents, there are myriad additional inclusions within bone; the ECM composition is 85%–90% collagen type I with the remainder including collagen types III and V,^[^ ^11^ ^]^ as well as over 200 other non‐collagenous proteins (NCPs) secreted by osteoblasts and osteoclasts.^[^ ^12^ ^]^ Bone composite is complex and difficult to visualise or understand holistically, as such, assumptions regarding its structure and formation were made historically.^[^ ^13^ ^]^ The advent of advanced nanoscopic 3D imaging techniques in recent years has substantially advanced knowledge of bone structure down to the atomic level.^[^ ^10^, ^14^ ^]^

**FIGURE 1:**
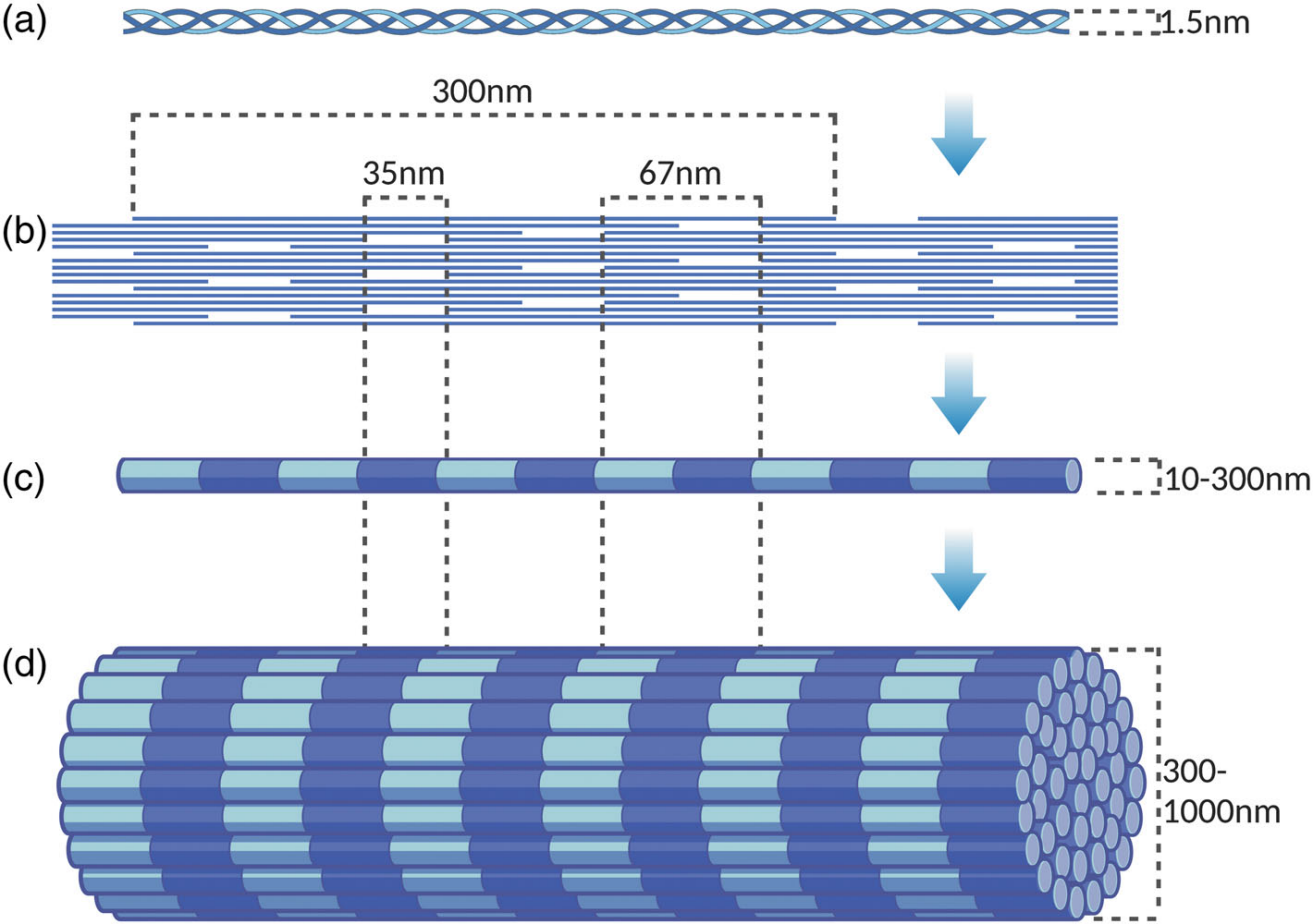
Diagram of collagen arrangement; (a) portion of collagen molecule; (b) 2D molecular organization within fibril; (c) 3D fibril with gap and overlap spaces aligned; (d) 3D fibre, with gap and overlap spaces aligned

While the macro‐structure of bone is highly variable and continuously remodelling, the ECM is hierarchically ordered and consistent (see Figure 1). Tropocollagen molecules are approximately 300 × 1.5 nm^[^ ^15^, ^16^, ^17^ ^]^ and are the basic subunit of collagen fibrils,^[^ ^12^ ^]^ which in turn form larger collagen fibres where collagen is aligned in 2‐dimensions; both tropocollagen and fibrils feature a quasi‐hexagonal arrangement.^[^ ^18^ ^]^ Fibrillogenesis occurs as per the enzymatic crosslinking of tropocollagen to form fibrils^[^ ^19^, ^20^ ^]^ following its secretion by osteoblasts (a lysyl oxidase process).^[^ ^21^ ^]^ Deamination at the molecular termini yields highly reactive aldehyde groups which covalently bond the molecules together^[^ ^17^ ^]^ via singular aromatic bonds.^[^ ^22^ ^]^ Neighbouring tropocollagen molecules are offset by ~67 nm^[^ ^12^ ^]^ in a staggered pattern,^[^ ^17^ ^]^ these repeated gaps form 35 × 1.5 nm helical grooves along fibrils.^[^ ^12^ ^]^ This pattern repeats laterally every five molecules, since the staggering occurs at 67 nm the gaps between molecules are 35 nm^[^ ^16^ ^]^ (i.e., 67 nm × 5 = 335 nm), these gaps are where HA crystals are able to nucleate.^[^ ^17^ ^]^ The organization of these mineralised fibres shift in gradients across the scales of observation causing variations in density, mechanical properties, and porosity.^[^ ^23^ ^]^

**FIGURE 2:**
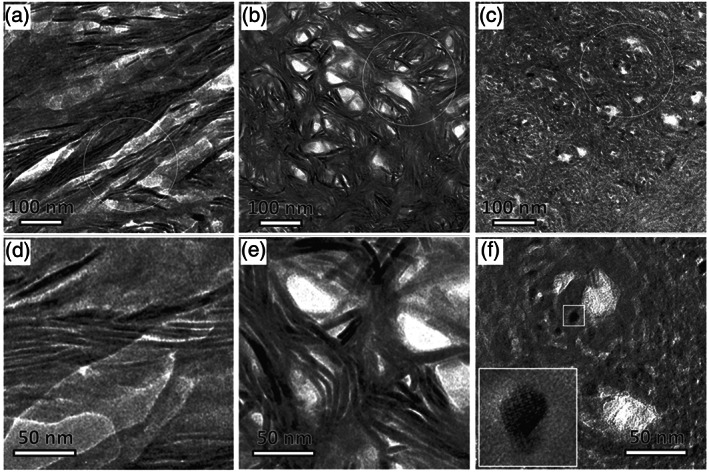
TEM scans showing three distinct bone patterns which are reprojections of one another when viewed at different angles; (a, d) Filamentous motif; (b, e) Lacy motif; (c, f) Rosette motif (Modified with permission—Copyright © 2018, Reznikov et al., The American Association for the Advancement of Science)

Multiple theories exist on the formation of collagen matrices, sometimes with only circumstantial evidence to support them, for example, bonding between collagen and bone/bioactive materials has been observed to occur in two ways (as demonstrated *in vivo* by Sautier et al.^[^ ^24^ ^]^): an afibrillar structure forms into which collagen fibrils are secured; or collagen fibres directly attach to the substrate surface. Additionally, some well‐established, yet incomplete, theories on bone mineral structure within bone matrix remain pervasive in literature.^[^ ^13^, ^25^ ^]^ Recent work utilising advanced combinations of high‐resolution tomography data in conjunction with 3D visualisation of ex vivo bone tissues has cast doubt over whether there is a distinction between intrafibrillar and extra‐fibrillar mineral phases^[^ ^26^ ^]^ (Figure 2). Transmission Electron Microscope (TEM) scans of ion‐milled bone samples revealed the extra‐fibrillar matrix (EFM) of bone to contain a continuous phase of HA crystallites bound together, and the ECM, by an array of NCPs.^[^ ^27^ ^]^

### 1.2 Hydroxyapatite structure and its orientation within the collagen matrix

**FIGURE 3:**
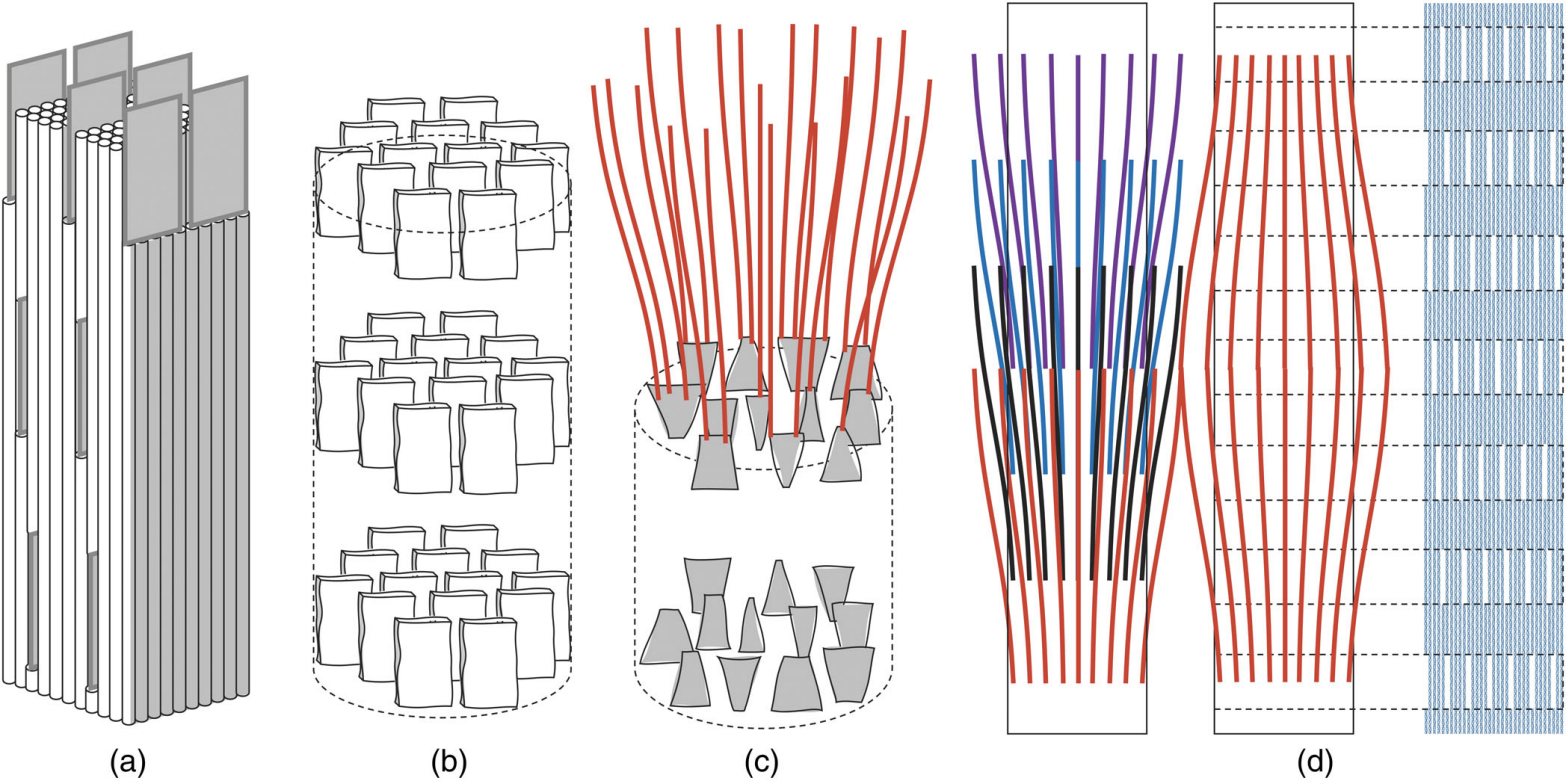
Comparison of old versus new theories on hydroxyapatite morphology within bone: (a, b) ‘Deck of cards’ mineral arrangement, with parallel plates occupying the intrafibrillar spaces; (c) slightly twisted plates in the intrafibrillar spaces with acicular crystals (red) projecting from them; (d) 2D illustration of the relationship between acicular crystals and fibrils

A ‘Deck of Cards’ analogy for minerals in bone was originally proposed by Weiner and Traub based on their electron micrographs of calcified turkey tendon.^[^ ^13^ ^]^ They proposed that all bone minerals are plate‐shaped and only appear needle‐like when observed off‐axis; additionally, these plates were considered to have a consistent, parallel organization relative to the observed fibril.^[^ ^28^ ^]^ This concept was illustrated as a stacked 2D structure while existing in a 3D space, which has been elusive to reproduce (see Figure 3a,b).

**FIGURE 4:**
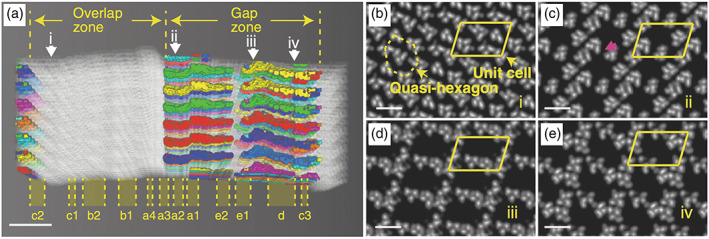
3D XRD electron density map of collagen structure. (a) Intermolecular voids within collagen structure, with gap/overlap zones labelled; (b–e) cross‐section slices along the collagen fibril at position i–iv, respectively, highlighted by white arrows in (a). (b) Typical structures of the overlap region. (c–e) 2–3 nm wide channels in gap region with varying cross‐section shapes. A unit cell is highlighted by a yellow parallelogram (Modified with permission—Copyright © 2020, YiFei Xu et al., Nature Communications)

In initial ex vivo observations, banding patterns were noticed when viewing the flat face of these plate‐shaped crystals^[^ ^29^ ^]^; current evidence points to these patterns being an artefact of the platelets forming via the coalescence of needle‐like HA crystals,^[^ ^10^ ^]^ though debates continue on this subject. This has been a limitation of observation techniques including Scanning Electron Microscopy (SEM) and Transmission Electron Microscopy (TEM) scans, which can only acquire 2D information on a surface, additionally, the preparation of samples for such analyses can cause structural changes and damage to the sample. In 2020 Xu et al.^[^ ^18^ ^]^ deduced via electron density mapping along with XRD analysis that the HA platelets are uniaxially orientated with regards to the collagen matrix (see Figure 4), twisting within the gap regions of collagen (see Figure 3c). These gaps are roughly cylindrical pores with an average diameter of 2 nm.^[^ ^18^ ^]^

HA crystals grow fastest in their *c*‐axis, which is what contributes to the commonly observed ‘needle‐like’ HA crystals; generally, such HA crystals are seen to have their *c*‐axis orientated in parallel with collagen.^[^ ^30^ ^]^ Since the growth of these crystals in any direction is halted by confinement, that is, contact with a boundary surface, it has been proposed that their alignment is likely the result of direct cellular control or sophisticated mediation with an array of NCPs that guide mineral propagation.^[^ ^31^ ^]^ However, this notion that the mature platelets contort and twist with the shape of their surroundings introduces the possibility that confinement might be a significant contributor to bone mineral morphology. HA alignment in collagen is, at least mechanically, dictated by competitive growth between crystals,^[^ ^18^ ^]^ those uninhibited in their *c*‐axis are the fastest growing, that is, growing longitudinally with collagen. The misaligned nuclei will halt development on contact with the neighbouring collagen and subsequently either be ionically reorientated to fuse with forming platelets, or resorbed due to Ostwald ripening.^[^ ^32^ ^]^ This is supported by experiments in which the transformation of ACP was studied in a *polymerised lyotropic liquid crystal* (PLLC) matrix. When confinement of Ca/PO was greater than 10 nm a variety of phases were produced during the maturation of the amorphous phase; however, when confined to less than 10 nm bone‐like HA platelets were consistently formed^[^ ^25^ ^]^; these small particle sizes are optimal for the toughness of bone.^[^ ^10^ ^]^

It is proposed through ex vivo analysis of bone samples that the intrafibrillar channels are nucleation zones for HA platelets they are not isolated, the amorphous mineral which initially inhabits these spaces runs continuously throughout the extra‐fibrillar space, making it a cross‐fibrillar phase.^[^ ^26^ ^]^ Crystallites which manage to form outside of these channels grow into curved acicular crystals, these also conform to their surroundings, and can simultaneously be intra‐ and extrafibrillar.^[^ ^10^ ^]^ Depending on the plane of observation the cross‐fibrillar acicular crystals present up to three fractal‐like motifs: filamentous, lacy, and rosettes.^[^ ^10^ ^]^ When viewed in‐plane the filamentous arrangement can be seen, mineral filaments gently curve longitudinally 50–150 nm across fibrils, typically extending beyond the 67 nm D‐period spacing of fibrils^[^ ^33^, ^34^ ^]^ (see Figure 2a,d). Out‐of‐plane angles reveal rosettes, which are hexagonal organizations of the same filaments seen extending across fibrillar spaces, slightly wrapping around multiple fibrils^[^ ^10^ ^]^ (as seen in Figure 2c,f). Isometric projections display lacy motifs, which appear as an intermediate of those previously described; 20–50 nm lens‐shaped gaps are characteristic which agree with them occupying extra‐fibrillar spaces^[^ ^33^, ^35^ ^]^ (see Figure 2b,e). Additionally, these filaments interact with HA platelets, though it is not understood whether the platelets nucleate the filaments or vice versa, but together they form a continuous, cross‐fibrillar array, figuratively sewing fibrils^[^ ^10^ ^]^ (see Figure 3c,d).

### 1.3 Physicochemical biomineralisation of bone

Biomineralisation is the process of living organisms producing hard parts or skeletons; these processes are controlled either by the environment or cellular processes within the organism itself.^[^ ^12^ ^]^ The former was dubbed as ‘biologically induced mineralisation’^[^ ^36^ ^]^ in 1981 by Lowenstam, and the latter ‘biologically controlled mineralisation’^[^ ^37^ ^]^ two years later by Mann. Both types of mineralisation require an aqueous environment to enable ionic agglomeration and arrangement, this is true in bone where water facilitates this process and helps to orientate HA platelets in the *c*‐axis.^[^ ^38^ ^]^ Water within bone is divided into three categories: bulk, bound, and structural water. Bulk water fills in larger gaps of the bone matrix such as the vascular network and aids in the transport of nutrients around bone tissue. Molecular dynamics (MD) simulations indicate that it also effectively acts as a collagenous lubricant,^[^ ^39^ ^]^ thus contributing to the viscoelastic properties of bone.^[^ ^40^ ^]^ Finite element simulations of the sub‐lamellar hierarchy of bone tissue suggested that bound water coats the surfaces of collagen molecules and HA crystals, potentially bonding more strongly to each than either component together.^[^ ^41^ ^]^ This water enhances the attraction between collagen and HA by forming salt bridges and hydrogen bonds between them.^[^ ^41^ ^]^ Finally, structural water is held within the lattice of HA phases, from amorphous to carbonated,^[^ ^42^ ^]^ this plays a key role in the established dissolution–re‐precipitation of ACP phases into HA by allowing ionic rearrangement throughout the mineral bulk, even in the absence of cells or external biological factors.^[^ ^25^ ^]^ Further, through large scale MD simulations Xu et al.^[^ ^43^ ^]^ showed that in the gap region of collagen, water density is approximately 30% lower, likely reducing the enthalpic penalty for mineral precursors to dissolve and precipitate.

As previously established, the biological apatite found within bone is highly substituted with carbonate which impacts all aspects of the mineral, particularly the morphological and mechanical characteristics. Carbonate in the crystal lattice of apatite results in the minerals having a three‐axis symmetry^[^ ^44^ ^]^ (hexagonal shape)^[^ ^45^ ^]^ via a shortening of the *c*‐axis,^[^ ^46^ ^]^ as opposed to the typical mirrored symmetry found in pure HA. Additionally, the crystal size is reduced, which in turn, decreases the lattice spacing,^[^ ^46^ ^]^ while increasing their relative surface area.^[^ ^47^ ^]^ The mechanical changes imparted from these structural differences include improved fracture toughness and diametral tensile strength,^[^ ^47^ ^]^ as well as a decrease in the elastic moduli which correlates with increasing carbonate content.^[^ ^46^ ^]^ The chemical composition of carbonated apatite is also known to increase from the stoichiometric HA ratio of 1.67 Ca:PO to as high as 2 Ca:PO,^[^ ^44^ ^]^ though this varies throughout apatite bulk. In fact, all of these aspects are known to vary irregularly, with disordered, hydrophilic domains covering the surfaces of highly crystalline regions.^[^ ^38^ ^]^ This variability in bone mineral has proved it to be exceedingly difficult to characterise; furthermore, the active biological influences which contribute to the continuous remodelling of apatite complexate this.

### 1.4 Biomineralisation mediated by native cells

**FIGURE 5:**
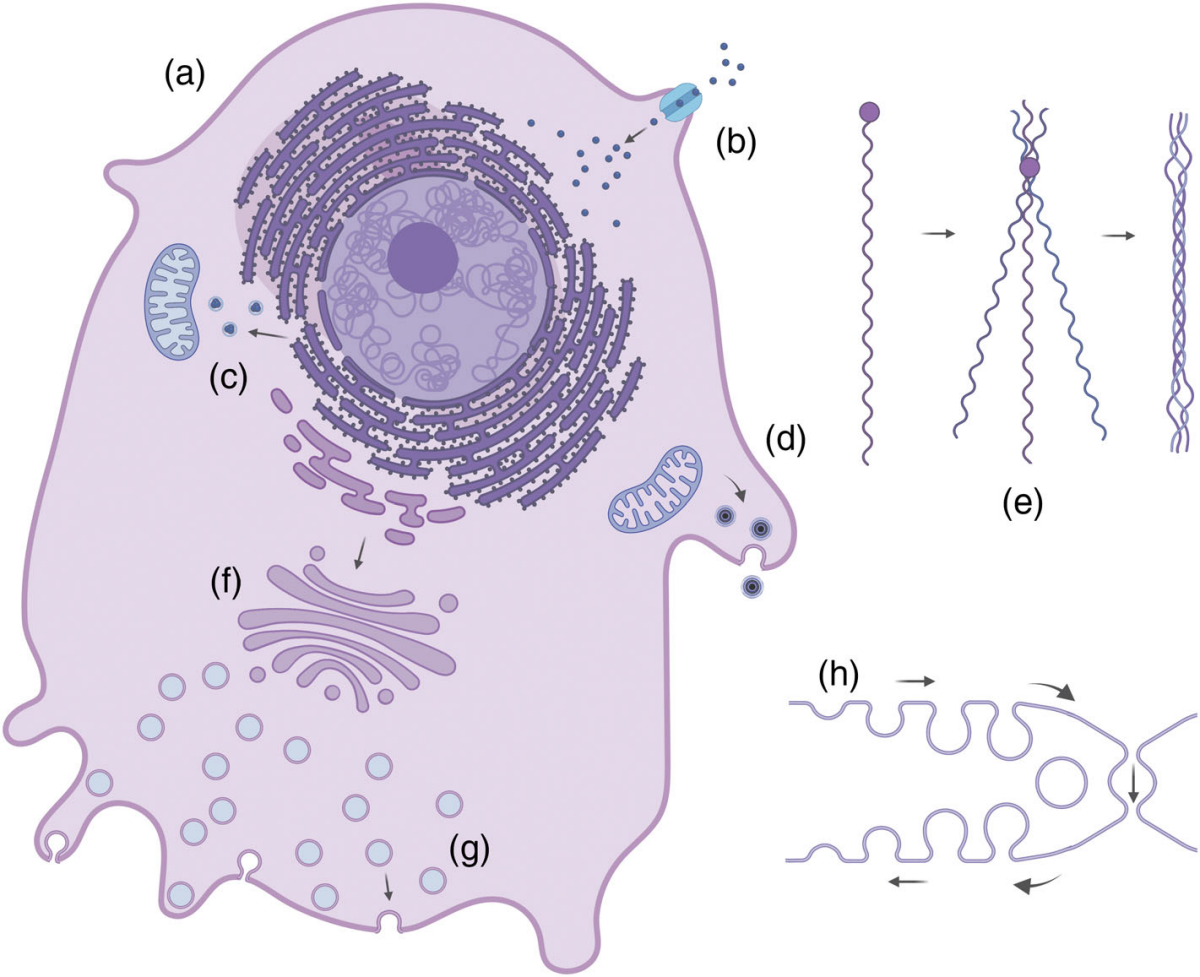
Osteoblast functions: (a) osteoblast; (b) intake of calcium ions towards the ER; (c) agglomerated calcium sent to the mitochondria for assembly with phosphate ions; (d) matrix vesicles containing ACP are secreted; (e) assembly of tropocollagen from procollagen; (f) transport from the ER to the Golgi complex; (g) exocytosis through cell membrane; (h) alternative exocytotic mechanism

Recent *in vitro* studies have suggested that all mammalian cells possess the ability to biomineralise given appropriate contact with ‘Calcium ions, phosphoester salt, and alkaline phosphatase’.^[^ ^48^ ^]^ These conditions allow for any somatic cell to nucleate stoichiometric HA precipitates that have 5–10 nm diameters.^[^ ^48^ ^]^ Indeed, using a two‐part Ca/PO mineralisation system (Ca solution + PO solution) real‐time liquid‐cell TEM analysis revealed that Ca/PO begins nucleating via particle attachment, rather than first forming amorphous phases,^[^ ^49^ ^]^ so somatic cells appear to facilitate this same behaviour internally. Though, to explain biomineralisation in bone, osteoblastic activity will be focused on the production of collagen, which is precisely coordinated with mineral distribution within these cells. As observed the confocal microscopy, the endoplasmic reticulum (ER) within osteoblasts is essentially where the building blocks of bone are assembled and mobilized.^[^ ^50^ ^]^ Procollagen is formed here and combined into tropocollagen before transportation to the Golgi complex, where these molecules are prepared for cellular export and used for fibrillogenesis.^[^ ^50^ ^]^ Through SEM and TEM Calcium ions have been observed to agglomerate and diverte to the mitochondria for ACP formation and release from the osteoblast (see Figure 5a–d), in the form of matrix vesicles.^[^ ^51^, ^52^, ^53^ ^]^

Based on studies, including one in which approximately 40,000 Matrix vesicles were examined, they have been shown to vary greatly in their size (10–200 nm, 100 nm average^[^ ^54^ ^]^) and composition (amorphous to fully crystalline^[^ ^55^ ^]^) depending on their maturity and proximity to the mineralisation fronts of bone.^[^ ^12^ ^]^ These vesicles become embedded in the ECM of nascent bone^[^ ^56^ ^]^ and may act as nucleation sites for further mineral development. Ex vivo human samples display the vesicles gradually rupturing as their amorphous contents crystallize,^[^ ^57^ ^]^ seeing HA needles penetrate through their surfaces, as found in mice.^[^ ^55^ ^]^ This can facilitate coalescence between vesicles^[^ ^58^ ^]^ into larger formations (490 ± 310 nm) known as calcospherulites^[^ ^59^ ^]^ in mice and rats, respectively. These have been shown in chicks to develop during periods of rapid bone growth and feature plate‐like crystal geometry.^[^ ^60^ ^]^ The evolution of ACP to crystalline HA, as seen in such particles, may occur independently of chemical interactions with other biological agents; *in vitro* study conducted by Lotsari et al.^[^ ^25^ ^]^ showed this to occur simply in the presence of water, where dissolution‐reprecipitation mechanisms enabled remodelling.

Both matrix vesicles and calcospherulites become embedded in bone, like osteoblasts when they transform into osteocytes; though little is known about how they are utilised^[^ ^61^ ^]^ despite there being evidence that they can be consumed over time.^[^ ^60^ ^]^ It is hypothesised that their purpose is primarily the sequestration of excess bone minerals for stoichiometric maturation,^[^ ^12^ ^]^ and perhaps to be later siphoned off via extracellular fluid. However, they could also act as reservoirs to sustain the long‐term function of osteocytes. Recent *in vivo* work by Gao et al.^[^ ^62^ ^]^ on mice has demonstrated that osteocytes can exchange mitochondria with each other via links in their cytoplasmic membranes, which effectively form a network. Such transfers help mitigate cellular stress by restoring ATP, oxygen, and reactive oxygen species' normal levels.^[^ ^62^ ^]^ Considering the above, it stands to reason that matrix vesicles and calcospherulites might be involved in this process, allowing for the distribution of crystalline HA throughout mature, living bone; sustaining the embedded osteocytes while simultaneously providing the materials needed to remodel and maintain healthy bone.

## 2 *IN VITRO* MINERALISATION OF DENSE COLLAGEN SCAFFOLDS

When seeded into a collagen scaffold with near physiological ECM density, stem cells set to work mineralising, and in some cases, modifying the matrix. This was found to occur in a study by Pedraza et al.^[^ ^63^ ^]^ wherein MC3T3‐E1 preostoblasts from mice were present in one such scaffold; here the cells simultaneously mineralised and contributed to the existing matrix with nascent collagen. Yet subsequent work conducted by the same research group (albeit with human dental progenitor cells) found that if the matrix is formed at a more physiological density, then fibrillogenic activity by the seeded cells is significantly reduced.^[^ ^64^ ^]^ Consequently, it can be assumed that this innately higher concentration would focus the cellular activity towards matrix mineralisation. However, as well as mineralisation, remodelling of the matrix will occur over time *in vivo*, as was found in the ex vivo examinations of subcutaneously injected dense collagen gels explanted from adult rats.^[^ ^65^ ^]^ As with collagen density, the more physiologically mineralised a dense collagen scaffold is, the better accommodating it will be to bone cells. An *in vitro* comparison of the activity and proliferation of MG‐63 human osteoblast‐like cells in mineralised and non‐mineralised dense collagen scaffolds found that the former saw deeper penetration and higher numbers of the cells within the substrates.^[^ ^66^ ^]^ Ultimately, cells will remodel a dense collagen environment until it is mineralised and has a matrix density like that of natural bone; by starting with a substrate which is already ideal, the local cell population can instead focus on integrating and optimizing the structure, thus accelerating the healing process.

The bone development process is not fully understood as per existing technical and technological limitations, therefore we may be able to glean further insight into its underlying mechanisms by attempting to synthesize bone *in vitro*.^[^ ^67^ ^]^ Below, a series of existing *in vitro* strategies and their physical‐chemical and biological barriers to achieving consistent and reliable mineralised collagen substrates that mimic bone tissue are summarised:

### 2.1 The use of Simulated Body Fluids (SBF) to promote *in vitro* mineralisation

SBF is a moniker for solutions with ion concentrations similar to human blood plasma that can precipitate apatite on osteoconductive material surfaces.^[^ ^68^ ^]^ A myriad of SBFs have been developed since their inception in 1990 (see Table 1), with their goal being to mimic blood plasma composition and pH whilst maintaining solution stability during preparation and use. The original SBF lacked the sulphate ions found in blood plasma, this omission was corrected in c‐SBF where these ions were introduced at a biomimetic concentration. Subsequently in 2003 Oyane et al.^[^ ^69^ ^]^ revised this formulation, developing r‐SBF, which directly matched the ionic composition of human blood plasma; however, undesirably it readily precipitated calcium carbonate due to the elevated levels of bicarbonate relative to prior SBF iterations.^[^ ^70^ ^]^ A year later Takadama et al.^[^ ^71^ ^]^ restored the bicarbonate concentrations to their original level, this new formula was dubbed n‐SBF. Round robin testing of c‐SBF versus n‐SBF found no differences in performance or reproducibility,^[^ ^72^ ^]^ therefore, both are recognised as an ideal solution for assessing bioactivity *in vitro* and precipitating apatite. Generally, n‐SBF is used as it more closely matches blood plasma, specifically in chloride content, where c‐SBF is approximately 43.4% higher.^[^ ^70^ ^]^ Beyond these standard formulations, highly concentrated SBFs were developed to accelerate the deposition of HA onto the surfaces of substrates. At higher concentrations, SBF is more of an inherited name, as the aspect of assessing the bioactivity of materials is superseded by mineralisation speed in priorities. These range from 1.5XSBF^[^ ^73^ ^]^ up to 10XSBF,^[^ ^74^ ^]^ a comparison between these two SBFs demonstrated that a 20 μm thick layer of apatite could be precipitated in just 2 h with 10XSBF versus 2 weeks with 1.5XSBF,^[^ ^74^ ^]^ a 168X reduction.

**TABLE 1:** Compositional comparison between human blood plasma and various SBF formulations (elements have been used for direct comparison across all variations since ionic species differ between certain SBFs)

	Elemental composition (mM)									
	Na	K	Mg	Ca	Cl	H	C	O	P	S
Plasma^[^ ^75^ ^]^	142.00	5.00	1.50	2.50	103.00	0.46	5.31	22.24	0.32	0.17
SBF^[^ ^68^ ^]^	142.00	5.00	1.50	2.50	148.80	0.08	0.83	3.97	0.32	0.00
c‐SBF^[^ ^72^ ^]^	142.00	5.00	1.50	2.50	147.80	0.08	0.83	4.30	0.32	0.17
r‐SBF^[^ ^69^ ^]^	142.00	5.00	1.50	2.50	103.00	0.46	5.31	22.24	0.32	0.17
n‐SBF^[^ ^71^ ^]^	142.00	5.00	1.50	2.50	103.00	0.08	0.83	4.30	0.32	0.17
1.5XSBF^[^ ^73^ ^]^	213.00	7.50	2.25	3.75	221.70	0.12	1.24	6.46	0.48	0.25
10XSBF^[^ ^74^ ^]^	398.03	2.62	0.60	6.82	622.81	1.27	1.43	18.85	2.58	0.00
10xSBF RA^[^ ^76^ ^]^	396.71	2.62	0.60	6.82	622.81	1.21	1.43	15.61	0.81	0.00

### 2.2 SBF‐induced surface mineralisation of dense collagen substrates

**FIGURE 6:**
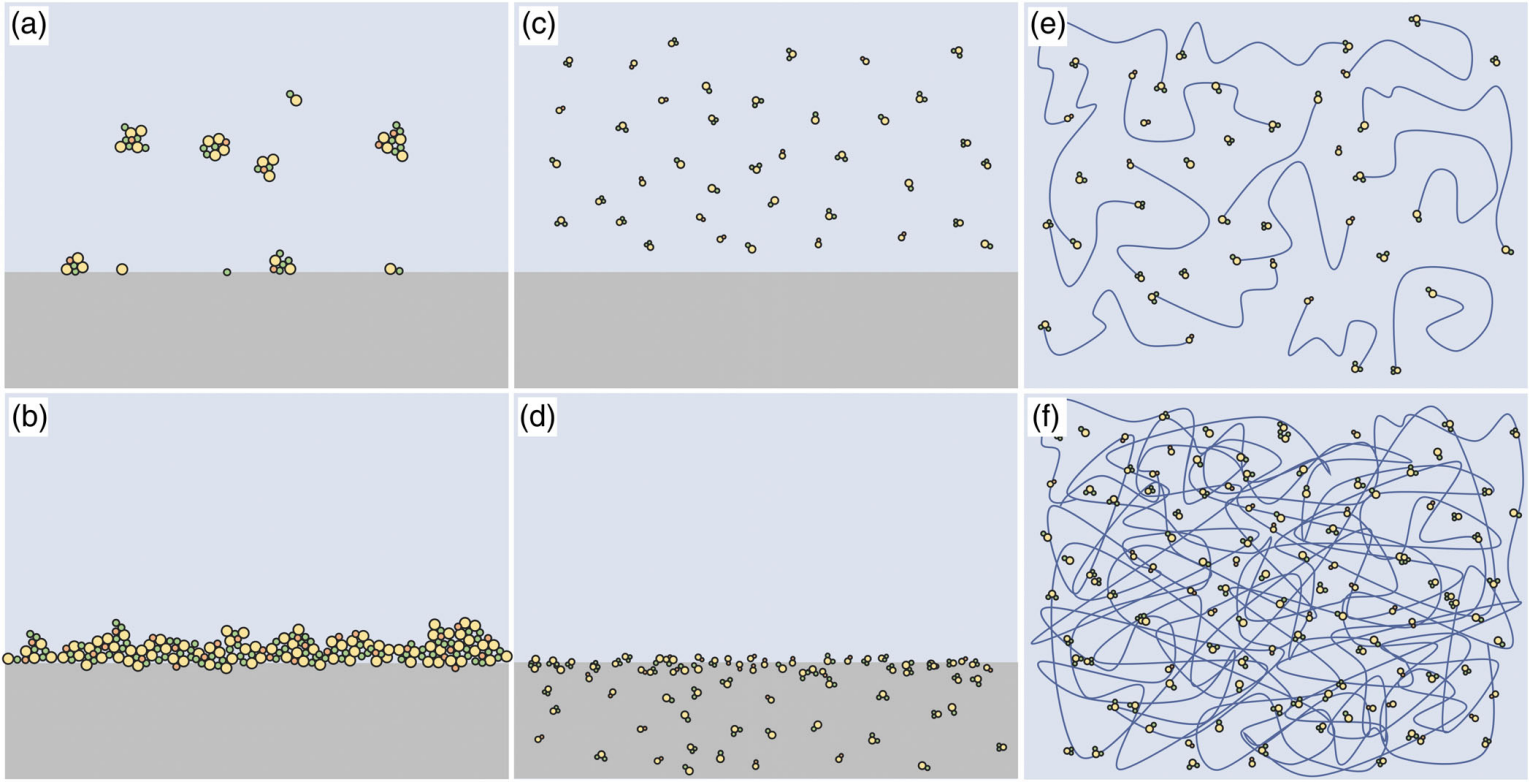
Comparison of collagen mineralisation methods: (a, b) surface mineralisation, with large cluster formation in solution and minimal substrate infiltration; (c, d) PILP method, with size‐limited mineral precursors in solution and deep substrate penetration through the surface; (e, f) hydroxyapatite/Collagen coprecipitation, showing nucleation on tropocollagen molecules before crosslinking, resulting in homogeneous collagen mineralisation

When dense collagen substrates are mineralised under SBF, they effectively undergo ‘biologically induced mineralisation’ rather than ‘biologically controlled mineralisation’. The latter is the *in vivo* process, whereas the former occurs as a reaction between the material surface and the environment, this is how, for example, crustaceans from their shells in the ocean.^[^ ^12^ ^]^ Herein lies the problem for this method of mineralising dense collagen substrates; regardless of the SBF iteration used or the exposure time, mineral predominantly accumulates on the surface, with little to no penetration^[^ ^73^, ^77^, ^78^ ^]^ (see Figure 6a,b). The minimal scaffold infiltration and virtual lack of intrafibrillar mineralisation can largely be explained energetically. SBF are supersaturated and possess enough kinetic energy to nucleate homogeneously within the solution, forming colloidal precipitates.^[^ ^79^ ^]^ Without the addition of limiting factors, these products will combine with other complementary ions and continue to grow^[^ ^78^ ^]^ beyond the intrafibrillar scale.^[^ ^80^ ^]^ Further, as the ions in the solution assemble, the energy of the system declines, that is, new nucleation sites become less likely to form on the material since they are ever less able to compete with the energetically favourable sites on pre‐formed nuclei. This issue cannot be circumvented by simply combining collagen with commercially available nano‐HA, a study by Yao et al.^[^ ^81^ ^]^ found only marginal surface mineralisation following 7 days of collagen‐HA contact; therefore, the ionic assembly of HA is key to its successful attachment to collagen. Despite seldom achieving intrafibrillar mineralisation, SBF has been shown to fully infiltrate and coat a macro‐porous collagen sponge through careful temperature management and pre‐treatment with calcium and phosphate solutions.^[^ ^82^ ^]^ This is a useful way of supplying collagen with apatite mineral for clinical use but does not produce a truly biomimetic scaffold and would likely serve best as a delivery mechanism for components to be reconstituted rather than a rapidly integrated and high‐performance bone tissue replacement.

### 2.3 Polymer‐induced liquid precursor

Collagen mineralisation in native tissues has been hypothesised to result from infiltration of a liquid‐phase mineral precursor,^[^ ^83^ ^]^ with biomineral precursors ~30 nm in diameter having been linked to the direct formation of intrafibrillar mineral.^[^ ^51^, ^83^ ^]^ ACP is found both intracellularly and extracellularly and has been observed crystallize over time^[^ ^60^ ^]^ and subsequently remodel into Carbonated HA crystals.^[^ ^83^ ^]^ A polymer‐induced liquid precursor (PILP) is a multi‐phase liquid in which ions, either through independent addition or SBF, are isolated into their own phase through the presence of select polymers which inhibit precipitation.^[^ ^83^ ^]^ PILP phases penetrate porous structures through minuscule openings by capillary forces acting on the phase boundaries within the liquid^[^ ^84^ ^]^ (see Figure 6c,d). *In vitro*, collagen treated with a Ca/PO‐PILP has been shown to remineralise, with the mineral phase evolving from ACP after 1 day to HA at 7 days, selected area diffraction (SAD) analysis confirmed ~95% mineralisation penetration throughout fibrils.^[^ ^81^ ^]^ *In vivo*, osteoporotic mouse tibias were Ca/PO‐PILP injected for 30 min, once every 4 weeks for 12 weeks total. Two hours post‐injection the Ca/PO‐PILP had fully infiltrated the bone tissue and after 8 weeks of treatment the osteoporotic group was like the healthy control group,^[^ ^81^ ^]^ showing great promise for remineralising living tissues.

Despite showing excellent results in select instances PILP is still in the early stages of development, particularly concerning bone repair. It requires precise control over the solution composition to maintain appropriate mineral cluster dimensions^[^ ^84^ ^]^ throughout treatment, most studies are continuous for days or weeks.^[^ ^85^ ^]^ In addition, the previously mentioned successes of collagen mineralisation via Ca/PO‐PILP were conducted on relatively small amounts of bone tissue; the *in vitro* sample was a cylinder of decellularized bovine bone tissue (<300 × 300 μm),^[^ ^86^ ^]^ and the *in vivo* study was performed on mouse tibia. While theoretically scalable, there are multiple complications to consider when adapting this process to larger‐scale applications.^[^ ^81^ ^]^ First, PILP mineralised collagen does not always display D‐spacing,^[^ ^85^ ^]^ instead saturating fibrils with minerals; though not necessarily detrimental, it is important to consider any effects of biomimetic deviation. The issue of ‘matrix congestion’ must also be considered, particularly for the remineralisation of living tissues (potentially PILPs greatest use case). Due to the infiltrative nature of PILP, entry points encounter more minerals than spaces deep within substrates,^[^ ^87^ ^]^ conceivably resulting in inhomogeneous mineralisation and possible inhibition due to pores becoming blocked.^[^ ^88^ ^]^ This cannot be accounted for in biologically derived tissues due to structural variability,^[^ ^87^ ^]^ further, 100 nm thick mineral‐based soap‐like bubbles of PILP formed in the attempted remineralisation of coral, spanning over larger pores.^[^ ^84^ ^]^ The implication is that matrix congestion could occur even with generous pore sizes. There is great potential for PILP as a method for mineralising/remineralising collagen, but further work is required to overcome its shortcomings. Currently, the inconsistency of results and the potential for unwanted structural changes to existing structures limit its utility.

### 2.4 Co‐precipitation of hydroxyapatite and collagen in SBF

In this process, soluble collagen is mineralised alongside fibrillogenesis, allowing for total and homogeneous coverage of HA onto the fibrils^[^ ^89^ ^]^ (see Figure 6e,f). Experiments utilizing this simultaneous assembly have been able to synthesize composites that, when assessed with X‐Ray Diffraction (XRD) and Nuclear Magnetic Resonance (NMR) spectroscopy, are quantitatively indistinguishable from natural bone (at comparably high collagen concentrations).^[^ ^90^ ^]^ In contrast to static or PILP mineralisation, intrafibrillar mineral formation is achieved without the need for additional components, which defies the previous understanding that NCPs are required.^[^ ^91^, ^92^ ^]^ Collagen can direct nucleation at the atomic level,^[^ ^87^ ^]^ even inhibiting the aggregation of ACP clusters like with PILP.^[^ ^80^ ^]^ Limiting cluster size not only increases the number of nuclei, but also the effective mineral surface area increases.^[^ ^80^ ^]^ Thus, soluble collagen accelerates and mediates the nucleation of HA in SBFs^[^ ^80^ ^]^; Wang et al.^[^ ^90^ ^]^ also demonstrated that at high saturations, collagen influences the orientation of HA precipitates without additives or cellular intervention. Since this form of *in vitro* mineralisation must occur before the formation of a dense scaffold, processes to control the resulting structure must be factored into any HA‐Col coprecipitation protocol. This could be advantageous given the right combination of methods but achieving a biomimetic combination of structural features from a collagen solution can be challenging. One such method which has been explored for tailoring the macro‐porosity (i.e., pore aspect ratio and relative alignment) of synthetic cancellous bone‐like scaffolds involves lyophilizing centrifuged collagen and crosslinking with EDC.^[^ ^93^ ^]^ HA‐collagen coprecipitation demonstrates the synergy between the two materials, when mixed, collagen accelerates and regulates the nucleation of HA without the aid of exogenous agents.

## 3 POTENTIAL IMPROVEMENTS TO ADDRESS EXISTING *IN VITRO* BIOMINERALISATION BARRIERS

**FIGURE 7:**
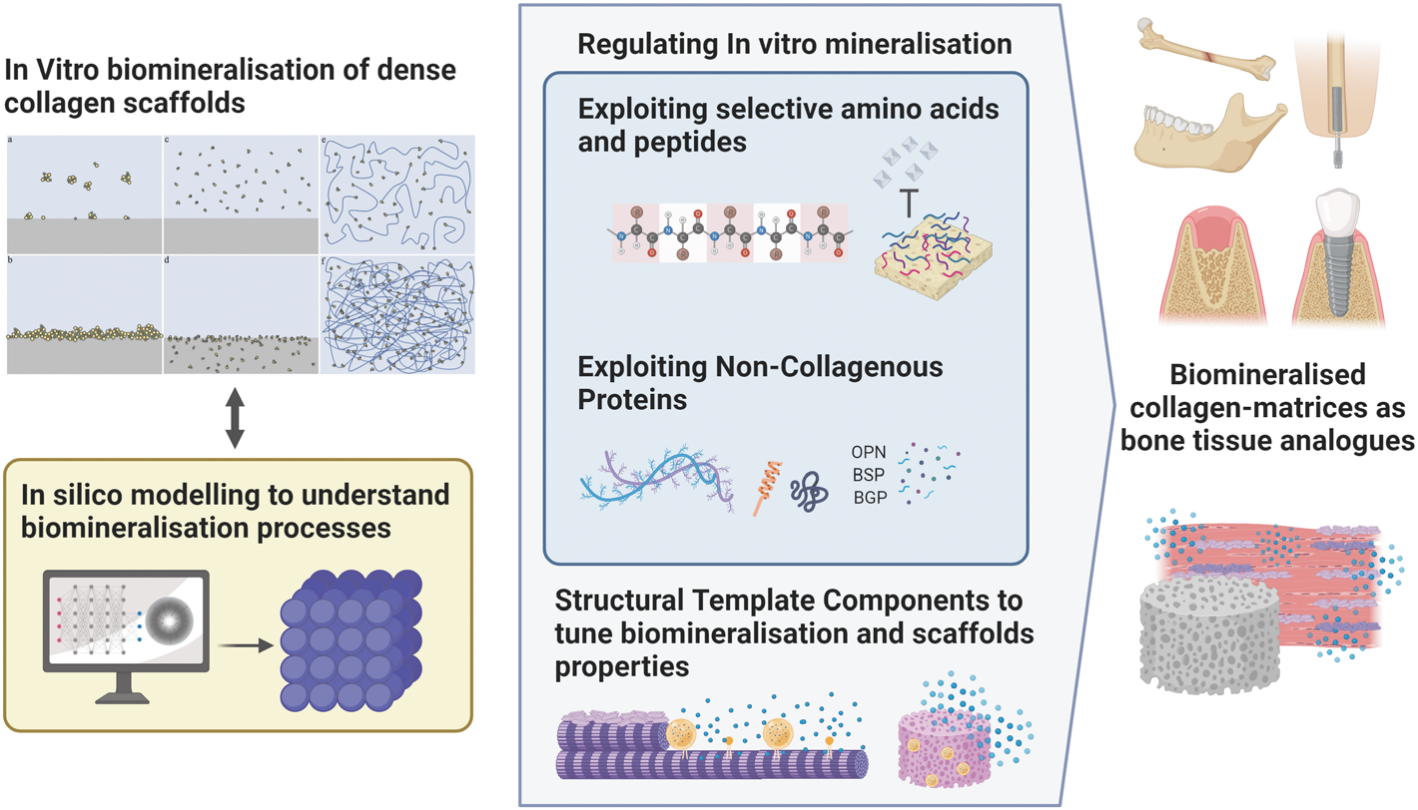
Illustration of potential improvement of existing in vitro biomineralisation methods towards creating biomineralized collagen matrices as bone tissue analogues: addressing existing knowledge gaps on biomineralisation processes and regulating mineralisation *in vitro* for improved *in vivo* tissue regeneration

The previously described processes for mineralising collagen show promise but all require further development due to shortcomings, below are descriptions of various additional components which may be useful in overcoming the barriers to *in vitro* mineralization (Figure 7):

### 3.1 Exploiting selective amino acids and peptides to regulate *in vitro* mineralisation

The integration of apolar amino acids (AAs) into collagen scaffolds may be useful in influencing collagen arrangement at the nanoscale. Amino acids are amines bonded to carboxylic acids (COOH) which also feature side chains, individual AAs are so small (~7 nm) that they are only able to affect singular HA crystal facets.^[^ ^94^ ^]^ Calcium ions, being positively charged, bind to carboxylated organic acid groups^[^ ^95^ ^]^ (functional groups), those which possess a negative charge (COOH in particular) are powerful promotors of HA precipitation. Both positively and negatively charged AAs are found in the gaps between collagen molecules where HA nucleates; both likely serve to draw in Ca^2+^ and PO_4_^3−^ ions,^[^ ^96^, ^97^ ^]^ and promote or inhibit the growth of select HA crystal faces.^[^ ^98^, ^99^ ^]^ Carmona and Rodriguez suggested that during HA formation, calcification may be enhanced by acidic residues scavenging protons which have been released from phosphate.^[^ ^100^ ^]^ Due to their stronger charge, Ca^2+^ and PO_4_^3−^ more easily adhere to the charged surface of collagen than OH ions.^[^ ^97^ ^]^ Depending on the charge of their side groups, AAs can have an increased preference to adsorb to HA^[^ ^101^ ^]^; some studies have found contradictory results^[^ ^102^ ^]^ albeit with indirectly comparable methodologies.^[^ ^103^ ^]^ Until recently the consensus in literature was that a stronger charge is likely to incur a greater inhibiting effect,^[^ ^98^, ^104^ ^]^ however, in 2021 Ecreg et al.^[^ ^103^ ^]^ concluded that the interactions between AAs and HA cannot be simplified as directly relating to their charge or polarity. For example, the interfacial energy barrier for the phase transition of Brushite to HA can be reduced by some acidic AAs^[^ ^105^ ^]^ and different functional groups can affect the Ca:PO ratio of HA precipitates: PO_4_ (1.67), COOH (1.49), NH_2_ (1.60),^[^ ^106^ ^]^ —COOH + —CO2— (1.35).^[^ ^107^ ^]^

Peptides are short amino acid chains and polypeptides are sequences of peptides. Larger polypeptides can affect multiple crystal facets across their length as well as with their side chains,^[^ ^108^ ^]^ enhancing their precipitator inhibiting effects. One such example is the Acidic serine aspartate‐rich MEPE‐associated motif (ASARM) which inhibits HA formation and growth when present in a gel diffusion system.^[^ ^109^ ^]^ Inhibiting mineralisation can be used to control mineral deposition, poly (aspartic acid) (PAsp) has been shown to inhibit surface mineralisation resulting in both extra‐fibrillar and intrafibrillar mineralisation within dense collagen scaffolds.^[^ ^78^ ^]^ Peptides which possess non‐polar terminals of alkyl or aromatic groups are likely to form assembled structures with high interfacial curvature as they are tapered; whereas those with a more uniform longitudinal profile are more likely to form flat structures.^[^ ^110^ ^]^

### 3.2 Exploiting NCPs to regulate *in vitro* mineralisation

Proteins are assemblies of polypeptides, aside from collagen they account for 10%–15% of bone matrix,^[^ ^111^ ^]^ and generally, they feature strong negative charges owing to their high amino acid content and phosphorylated residues.^[^ ^87^ ^]^ Their complex and modular structures,^[^ ^41^ ^]^ typically consisting of between 180 and 200 different molecules,^[^ ^112^ ^]^ permit NCPs to attract Ca^2+^ and PO_4_^3−^ ions in a controlled manner, such that the resulting local supersaturation is conducive to optimal HA nucleation.^[^ ^31^, ^113^ ^]^ These molecules can be both promoters and inhibitors of nucleation depending on their relationship with the surrounding environment, unbound inhibitors can prevent the growth of multiple crystal faces, whereas when adhered to a surface they add to the nucleation sites. NCPs within the bone can be grouped into three main categories: glycoproteins, proteoglycans, and acidic Gla proteins.^[^ ^12^ ^]^ The former two are found in mineralized tissues across phyla, whereas the latter is specific to bone and dentin within vertebrates.^[^ ^114^ ^]^ Acidic glycoproteins (as shown in Table 2) can strongly inhibit crystal nucleation when dispersed within a fluid, whereas when adhered to a substrate they powerfully induce nucleation,^[^ ^115^ ^]^ this can partly be explained by the stability introduced when a particle adheres to a solid surface, with much less free movement particles can cluster together to form a ‘critical nucleus’.^[^ ^12^ ^]^

**TABLE 2:** Bone‐related glycoproteins used to regulate *in vitro* mineralisation

Glycoprotein	Role	*In vivo*	*In vitro*
Osteopontin (OPN)	Mostly present in mineralised tissues.^[^ ^116^, ^117^ ^]^ Links areas of bone formed at different times.^[^ ^118^ ^]^ Inhibitory to mineral formation.^[^ ^119^ ^]^	Knockout mice had fewer, larger HA crystals,^[^ ^120^, ^121^ ^]^ and lower collagen/HA synthesis.^[^ ^122^ ^]^	OPN binds to HA crystals, inhibiting formation *in vitro*.^[^ ^123^ ^]^
Bone Sialoprotein (BSP)	Specific to mineralised tissues.^[^ ^124^ ^]^ Aids ACP nucleation and crystallisation.^[^ ^125^ ^]^ Enhances mineralisation when bound to collagen.^[^ ^109^ ^]^	A mouse defect model saw better integration and increased bone volume with BSP‐coated scaffolds.^[^ ^126^ ^]^	Significantly increases Ca/PO uptake in an agarose gel system.^[^ ^6^ ^]^
Bone Morphogenetic Protein‐2 (BMP‐2)	Osteoinductive growth factor, US‐FDA approved for use in collagen sponge.^[^ ^127^ ^]^	Spinal defects in rats were successfully mended with BMP‐2 functionalized porous scaffolds.^[^ ^128^, ^129^ ^]^	BMP‐2‐loaded scaffolds improved cell proliferation, leading to higher OCN and OPN expression.^[^ ^130^ ^]^

Aside from various biological functions proteoglycans are proteins responsible for managing hydration within extracellular matrices.^[^ ^41^ ^]^ The surfaces of collagen fibrils are coated with small leucine‐rich proteoglycans (SLRPs) which keep the fibrils hydrated by adhering water molecules to the surface.^[^ ^131^ ^]^ During fibrillogenesis, once the collagen has crosslinked the hydration layer becomes trapped and experiences kilopascals of pressure^[^ ^132^, ^133^, ^134^ ^]^; this pressure is translated towards nucleating HA crystals which over time displace the trapped fluid.^[^ ^135^ ^]^ This controlled presence of extracellular water is indicated to manage optimal mineralisation throughout bone development.^[^ ^136^ ^]^ Even in mature bone, tight associations between water and HA crystals are responsible for their long‐order arrangement and aspect ratios.^[^ ^10^ ^]^ Mechanically these bonds enhance energy dissipation in bone by acting as plasticizers,^[^ ^137^ ^]^ where otherwise crystalline fractures might occur under strain.

Gamma‐carboxyglutamic (Gla) proteins are generated by post‐translational modifications of glutamate residues following the carboxylation of vitamin K,^[^ ^112^ ^]^ the two involved with the bone are Matrix Gla Protein (MGP) and Osteocalcin (OCN), AKA Bone Gla Protein (BGP).^[^ ^138^ ^]^ The carboxylic acid residues within these proteins have a high affinity for binding to Calcium, chelating Ca^2+^ ions between two of them.^[^ ^112^, ^138^ ^]^ MGP is described as inhibitive to biomineralisation and is used to regulate calcification in various soft tissues,^[^ ^138^ ^]^ for example, preventing mineral formation in the vascular networks of bone.^[^ ^139^ ^]^ Osteocalcin preferentially binds to the HA crystals in bone^[^ ^140^ ^]^ as well as those which are synthetically produced^[^ ^12^ ^]^ and acts as both a promoter and inhibitor of mineralisation.^[^ ^138^ ^]^ When free in solution OCN will efficiently accrue Calcium and limit the formation of Ca/PO clusters,^[^ ^12^ ^]^ but this also allows OCN to deliver large quantities of Calcium when functionalizing the ECM of bone, thus aiding mineralisation where it is applied.

While not specifically relating to bone, NCPs which concern the mineralisation of enamel and dentin also interact with apatite minerals and could be utilised in synthetic scaffolds for bone repair. Amelotin is a relatively recently discovered protein^[^ ^141^ ^]^ which sees peak expression when dental enamel is maturing.^[^ ^142^ ^]^ One of its comprising peptide sequences Ser‐Ser‐Glu‐Glu‐Leu (SSEEL) has recently been found to have a potent effect on HA development *in vitro*.^[^ ^143^ ^]^ Zhang et al.^[^ ^144^ ^]^ found that phosphorylated SSEEL bound to ACP particles, drawing the internal Ca^2+^ ions to the surface; whereas, non‐phosphorylated SSEEL stimulated the dissolution‐reprecipitation mechanism of HA by chelating Ca^2+^ ions found at the surface of acidic ACP regions.^[^ ^144^ ^]^ This protein effectively moderates the crystallisation of HA by reducing the nucleation rate of apatite, while also boosting ionic exchange between amorphous phases.^[^ ^143^ ^]^ For more information on phosphorylated proteins and their influence on apatite development, it is recommended to read the extensive and detailed review by George and Veis (2008).^[^ ^31^ ^]^

### 3.3 Exploiting structural template components to tune biomineralisation and scaffolds properties

While the quantity, quality, and placement of HA can have a morphological impact on the bone structure at the nanoscale, macroscopic alterations to the bone matrix can be induced by combining collagen with *structural template components*. These may be foreign or native to bone tissues and can have a profound effect on the hierarchical organization of collagen and the mechanical properties of engineered scaffolds. ‘Hypermineralised’ whale bones have recently been found to contain lipid‐rich phases which contribute to extremely high bone mineral content, up to 95%.^[^ ^145^ ^]^ Such high mineral content is encouraged by the ionic inclination of Ca^2+^ to bind to PO_4_^3−^. By integrating phospholipids into the collagenous framework there is a greater number of nucleation sites which enable more mineralisation than with collagen alone.^[^ ^145^ ^]^ Phospholipids are found in human bone marrow, but only trace amounts are detected in the mineralised matrix of bone,^[^ ^146^ ^]^ nevertheless, their inclusion in an engineered scaffold would be biomimetic. Perhaps collagen‐lipid complexes could be used as a brute‐force strategy to dramatically increase the mechanical properties of *in vitro* synthesized bone substrates. In addition, polysaccharides like Chitosan can be used in conjunction with collagen to produce dense gels which bear similarities to osteoid.^[^ ^147^ ^]^

Silk fibroin, when used as a template for HA mineralisation, can orientate and alter the formation of HA crystals.^[^ ^148^ ^]^ HA crystals align their *c*‐axis with the silk fibrils in the same manner which is observed when mineralized between collagen fibrils.^[^ ^149^ ^]^ With increased silk fibroin content, the morphology of crystals moves from the typically ‘needle shape’ *in vitro* HA crystal to a more ‘rice‐like’ structure.^[^ ^149^ ^]^ This shortening along the *c*‐axis or broadening of the crystals is a step towards a more biomimetic crystal, though precipitates can tend to form with higher calcium content than in stoichiometric apatite.^[^ ^150^ ^]^ Silk fibroin features an affinity for HA crystals like that of collagen, when assembled together they have produced scaffolds with ordered and consistent secondary structures. Additionally, osteogenic differentiation of MSCs occurred after exposure to these hybrid substrates,^[^ ^151^ ^]^ showing their suitability for cell encapsulation, although lower proliferation was seen compared to collagen controls.^[^ ^150^ ^]^ Marelli et al.^[^ ^152^ ^]^ found that incorporating a processed form of silk fibroin into a crosslinked collagen gel resulted in a 9X increase in the compressing modulus of gels.

### 3.4 The use of in silico modelling to understand biomineralisation processes

Some components of bone are remarkably consistent in their make‐up and interactive properties. For example, the ions which comprise HA, that is, Ca^2+^, PO_4_^3−^, and OH^−^, can easily be modelled due to their simplicity and regularity,^[^ ^153^ ^]^ even collagen can be reliably modelled owing to its precise structure. While Type I collagen is a complex molecule, the amino acid subunits which assemble to make them are not,^[^ ^154^ ^]^ so despite the computational complexity encountered from having many interactive components, simulations of collagen, ions, and water^[^ ^41^ ^]^ can produce trustworthy data with finer detail than could be directly observed with conventional means. Xue et al.^[^ ^154^ ^]^ were able to provide evidence that small Ca/PO clusters can form in solution before attaching to the surface of collagen. These clusters favour nucleating around charged residues and prefer sites where the amino acids Glutamine and Arginine both occur. They concluded that the morphology of ACP clusters could be dictated somewhat by the region of collagen onto which they attach.^[^ ^154^ ^]^

Unfortunately, challenges arise in modelling the development of bone due to the highly substituted nature of HA, as well as the inherent variability of bone tissue when considering beyond the atomic and nanoscale. As previously established, apatite within the ECM of bone is continuous across difference domains,^[^ ^10^ ^]^ containing stoichiometrically crystalline domains and ionically deficient species^[^ ^10^ ^]^ which intermingle simultaneously with salts and AA complexes, and so forth.^[^ ^41^ ^]^ These aspects obfuscate crystallographic analysis, causing widespread disagreement between published data.^[^ ^153^, ^155^, ^156^, ^157^, ^158^, ^159^ ^]^ Therefore, approximations and assumptions typically must be used when simulating bone minerals^[^ ^10^, ^41^ ^]^; even when these data are obtained experimentally, they are seldom applicable to other studies. These issues are a substantial barrier to validating in silico models of bone,^[^ ^160^ ^]^ though modelling has already provided unique insight into the atomic and molecular interactions at the early stages of bone formation.^[^ ^154^ ^]^ Despite the challenges in validating in silico bone simulations, isolated models of molecular interactions between collagen molecules^[^ ^154^ ^]^ and complementary structural agents are feasible and could accelerate the development of novel matrices which better mimic cancellous or cortical bone structure.

## 4 OVERLOOK ON TRANSLATING MINERALISED COLLAGENOUS SCAFFOLDS TOWARDS CLINICS

Collagen matrices with higher densities (~250 mg/ml), and concurrently higher mechanical properties, have proven to resist precipitous resorption and consequently perform better when implanted in the abdominal tissues of rats.^[^ ^161^ ^]^ In the past 5 years there has been increased focus on testing scaffolds in animal models, which utilise coprecipitated collagen–hydroxyapatite, typically in conjunction with a synthetic polymer for increased mechanical properties and structural control.^[^ ^162^, ^163^ ^]^ Zhang et al.^[^ ^164^ ^]^ mineralised collagen through coprecipitation which they dried and powdered before combining with a Chitosan solution to cast into a film, the film was then backed by electrospun PCL/PVP nanofibers loaded with berberine (a herbal monomer). The efficacy of this bilayer implant was like that of an un‐named, commercially available mineralised collagen membrane when implanted in rat femurs. Zhang et al.^[^ ^165^ ^]^ implanted titanium screws at femoral wound sites of rats, either directly into bone, or into an extra‐fibrillary (EMC) or intrafibrillary (IMC) mineralised collagen scaffold. The IMC group saw mechanical properties only slightly lower than the direct implant but had markedly better bone regeneration than others, while the EMC group performed poorly in both regards. Other than coprecipitated matrices, PILP injections have been used to improve the stability of titanium implant screws in osteoporotic bone tissues of ovariectomized rats, generating similar levels of implant‐bone contact to that seen in the control group with healthy bone.^[^ ^166^ ^]^

### 4.1 Existing clinical applications and barriers

Human trials of hydroxyapatite‐collagen composites are somewhat limited due to the narrow range of approved, lab‐produced materials; with most taking place in China where a coprecipitated mineralised collagen powder‐PLA hybrid material, developed in 2004 by Liao et al.^[^ ^167^ ^]^ was sanctioned as a class III medical device.^[^ ^168^ ^]^ This material has been used for calcaneus void filling where no rejection or necrosis occurred in any subjects^[^ ^168^ ^]^; vertebral fusion where the hybrid substrate achieved 95.7% fusion versus 100% in autograft^[^ ^169^ ^]^; and to aid the attachment of locking plates to the distal radius,^[^ ^170^ ^]^ in which ulnar variance was increased in groups without mineralised collagen‐PLA but otherwise no functional differences were detected. All the above implants were accompanied by metal plates or support structures therefore, alone, these scaffolds are not suitable for bone repair.

### 4.2 Commercially available mineralised collagen‐based products

Bongold™ is a patent‐protected composite of type I collagen and synthetic hydroxyapatite^[^ ^171^ ^]^ which becomes both osteogenic and osteoinductive when assimilated with bone marrow aspirate from the host.^[^ ^172^ ^]^ The mineral content of this product is approximately half of what is found in natural bone and does not contain carbonate substitutions.^[^ ^173^ ^]^ It is marketed solely as a cancellous bone void filler and is distributed in the form of cylinders, blocks, and particles to accommodate various cavities; Bongold™ is alternatively marketed as HEALOS®, OssiMend®, MASTERGRAFT®, and Vitoss®.^[^ ^171^ ^]^ This product line has been demonstrated to be a suitable alternative to autograft in the internal fixation of metal implant devices.^[^ ^174^ ^]^ SynOss is another notable Ca/PO‐collagen composite material which is distributed in the same forms as Bongold™ and is targeted toward Dentistry. The compositions of the two products differ in that while both contain synthetic apatite, SynOss mineral is carbonated and present at nearly double the concentration (80% vs. 45%).^[^ ^171^, ^175^ ^]^ Despite having a closer composition to bone than Bongold™ there are reports from *in vivo* investigations that when used as a scaffold, SynOss was in most cases unable to induce bone regeneration (however, it should be noted that clotting had likely occurred prior to implantation).^[^ ^176^ ^]^ The existing void‐filling products highlight the importance of further developing analogous bone tissues, as where they have been successfully implemented, for example, vertebral fusion, the rates of osseointegration and near‐total lack of rejection or necrosis are class leading.^[^ ^168^, ^169^, ^174^ ^]^ A major issue with these products is that they are seldom used alone, most often being fixative aids for metallic plates, and so forth.^[^ ^174^ ^]^ Ideal bone repair substrates would have enough strength to function alone, perhaps accompanied by a degradable cement or support instead of metal.

## 5 CONCLUSIONS

Very few alternatives to autologous bone grafts are available for clinical use and, contrary to the marketing of some commercially available bone void fillers, a true bone substitute is yet to be developed.^[^ ^163^ ^]^ Bone is a highly complex composite and mimicking its properties and structure necessitates the introduction of numerous components which adds variables to any engineered scaffold, this presents a barrier to development, especially considering the high cost of collagen. Achieving high mechanical properties in collagen based scaffolds, through physiological‐like ECM density and intrafibrillar mineralisation, is arguably essential to avoid collagenolytic digestion.^[^ ^177^ ^]^ Existing strategies to combat this issue have involved the use of harsh chemical crosslinkers to improve the stability of collagen substrates (e.g., glutaraldehyde and formaldehyde).^[^ ^178^ ^]^ Unfortunately, these cytotoxic chemicals are counterproductive to the rapid healing qualities which collagen should normally provide as initially they increase inflammation and inhibit cellular infiltration.^[^ ^179^ ^]^ Scaffolds must be remodelled in situ by cells for true integration, that is, embedding of osteocytes,^[^ ^62^ ^]^ introducing vasculature,^[^ ^139^ ^]^ and restructuring the ECM to suit the implant site.^[^ ^165^ ^]^ The more an implant resembles the resultant osseous structure, the faster the integration will be as less time and energy must be devoted by local cells, consequently recovering faster. The work by Zhang et al.^[^ ^165^ ^]^ demonstrates this, where an intrafibrillary mineralised scaffold greatly outperformed one which was extra‐fibrillary mineralised, both mechanically and in healing rate. While all mineral content within the ECM of bone contributes to its mechanical strength, the intrafibrillar mineral has the most substantial effect, owing to the rigidity imparted by intrafibrillar hydraulic pressure.^[^ ^132^, ^133^, ^134^ ^]^

Biomimetic approaches to synthesizing bone are invaluable in enhancing our understanding of the continually elusive mechanisms behind bone development, however, these approaches might not provide the most direct path toward producing clinically available synthetic bone. For example, the current consensus is that collagen is most likely mineralised through a PILP process,^[^ ^83^ ^]^ and while effective, *in vitro* coprecipitation methods have been shown to achieve results which at least match those of PILP.^[^ ^90^, ^91^, ^92^ ^]^ Perhaps a combination of hydroxyapatite‐collagen coprecipitation and the PILP method might prove highly efficient in biomimetically mineralising analogous substrates, as coprecipitation is consistently able to deposit intrafibrillar minerals,^[^ ^89^, ^90^ ^]^ and PILP is effective as saturating extracellular matrices with ACP.^[^ ^81^ ^]^ This would allow collagen to mediate the deposition of HA crystallites on its surface and molecular end termini, that is, intrafibrillar spaces. Following a form of densification, such as plastic compression,^[^ ^180^ ^]^ the mineral seeded collagen matrix could be infiltrated with ACP via the PILP method. By preemptively installing orientated crystallites between collagen molecules the evolution of the amorphous extracellular phase into a continuous,^[^ ^18^ ^]^ fractal‐like, multiphasic mineral network^[^ ^10^ ^]^ could be accelerated. Additionally, the coprecipitation stage could be exploited to introduce components to alter the resulting matrix,^[^ ^109^, ^110^, ^147^, ^152^ ^]^ and/or embed growth factors or release agents such as drugs.^[^ ^164^ ^]^

## CONFLICT OF INTEREST

The authors declare no conflicts of interest.

## Data Availability

The data that support the findings of this study are openly available in Newcastle University at https://data.ncl.ac.uk.
